# Altered mechanisms of genital development identified through integration of DNA methylation and genomic measures in hypospadias

**DOI:** 10.1038/s41598-020-69725-1

**Published:** 2020-07-29

**Authors:** Melissa A. Richard, Pagna Sok, Stephen Canon, Wendy N. Nembhard, Austin L. Brown, Erin C. Peckham-Gregory, Minh Ton, Erik A. Ehli, Noah A. Kallsen, Shanna A. Peyton, Gareth E. Davies, Ashay Patel, Ismael Zamilpa, Charlotte A. Hobbs, Michael E. Scheurer, Philip J. Lupo

**Affiliations:** 1 Department of Pediatrics, Baylor College of Medicine, Houston, TX USA; 2 Arkansas Children’s Hospital, Little Rock, AR USA; 3 Department of Urology, University of Arkansas for Medical Sciences, Little Rock, AR USA; 4 Department of Epidemiology and Arkansas Center for Birth Defects Research and Prevention, Fay W. Boozman College of Public Health, University of Arkansas for Medical Sciences, Little Rock, AR USA; 5 Avera Institute for Human Genetics, Sioux Falls, SD USA; 6 Department of Pediatrics, College of Medicine, University of Arkansas for Medical Sciences and Arkansas Children’s Research Institute, Little Rock, AR USA

**Keywords:** Hypospadias, DNA methylation

## Abstract

Hypospadias is a common birth defect where the urethral opening forms on the ventral side of the penis. We performed integrative methylomic, genomic, and transcriptomic analyses to characterize sites of DNA methylation that influence genital development. In case–control and case-only epigenome-wide association studies (EWAS) of preputial tissue we identified 25 CpGs associated with hypospadias characteristics and used one-sample two stage least squares Mendelian randomization (2SLS MR) to show a causal relationship for 21 of the CpGs. The largest difference was 15.7% lower beta-value at cg14436889 among hypospadias cases than controls (EWAS P = 5.4e−7) and is likely causal (2SLS MR P = 9.8e−15). Integrative annotation using two-sample Mendelian randomization of these methylation regions highlight potentially causal roles of genes involved in germ layer differentiation (*WDHD1, DNM1L, TULP3*), beta-catenin signaling (*PKP2, UBE2R2, TNKS*), androgens (*CYP4A11, CYP4A22, CYP4B1, CYP4X1, CYP4Z2P, EPHX1, CD33/SIGLEC3, SIGLEC5, SIGLEC7, KLK5, KLK7, KLK10, KLK13, KLK14*), and reproductive traits (*ACAA1, PLCD1, EFCAB4B, GMCL1, MKRN2, DNM1L, TEAD4, TSPAN9, KLK* family). This study identified CpGs that remained differentially methylated after urogenital development and used the most relevant tissue sample available to study hypospadias. We identified multiple methylation sites and candidate genes that can be further evaluated for their roles in regulating urogenital development.

## Introduction

Hypospadias is a common birth defect in boys characterized by the urethral opening forming on the ventral side of the penis rather than at the meatus. Hypospadias may further be characterized by the presence or absence of chordee, a curvature of the penis resulting from fibrous tissue and skin tethering. The etiology of hypospadias is incompletely understood, but genetics and environmental factors have been implicated in its development^1^. Emerging evidence suggests that genetic and environmental factors involved in the etiology of hypospadias may be heterogeneous by severity^2–5^. Therefore, clinical characteristics, including chordee and severity, should be considered to identify developmental pathways that lead to hypospadias.

The differentiation of a single-cell zygote into a complex, multicellular organism is controlled in part through dynamic epigenetic modifications that regulate gene transcription during embryogenesis^6^. The genital tubercle forms as an undifferentiated outgrowth that sexually differentiates into an anlage for either the male penis or female clitoris at 8–14 weeks of gestation. During sexual differentiation, androgens in a male fetus signal fusion of the urethral folds that form what will become the urethra. DNA methylation is one epigenetic mechanism that could regulate the transcription of developmental genes. Methyl groups are added to cytosine-guanine (CpG) dinucleotides in regulatory regions of the genome and can affect the transcription of DNA without altering its sequence variation. Genomic methylation is a dynamic state that can be influenced by the underlying sequence variation and altered by environmental exposures. Studies have also shown that altered DNA methylation can be inherited through generations to affect health risk despite the complete epigenetic reprogramming of embryonic germ cells^7^.

Candidate gene approaches for hypospadias have focused on genes related to sex hormones^8^ and development of the genital tubercle^9^. However, genome-wide association studies (GWAS) of hypospadias do not replicate variation in these candidate genes at the genome-wide level (P < 5e−8), rather they have identified other genomic regions that do not fully explain hypospadias risk^10^. Environmental exposures and maternal metabolic traits have been widely assessed for association with hypospadias^2,3,11,12^, which may also affect DNA methylation. Thus, DNA methylation may serve as a functional link between the influence of genes and environment on human disease and is of particular interest for its role in regulating human development. One previous epigenome-wide association study (EWAS) by Choudhry et al. identified 14 CpGs associated with hypospadias (P < 1e−4) among a sample of 12 affected boys and 8 boys without hypospadias^13^. After correction for multiple testing, Choudhry et al. identified methylation within *SCARB1* and *MYBPH* as significantly associated with hypospadias, but these findings have yet to be replicated in larger samples. Additionally, no study to date has used an epigenome-wide approach to identify CpGs differentially methylated in relationship to chordee or hypospadias severity.

We sought to characterize methylation among moderate to severe cases of hypospadias using an integrative analytic approach. We first conducted epigenome-wide association studies for hypospadias (case–control), chordee (case-only), and severity (case-only). Genetic determinants were estimated for CpGs suggestively associated with hypospadias characteristics. We then used genetic instrumental variable analysis for differentially methylated CpGs and genes within regions of differential methylation to characterize causal relationships in the etiology of multiple features of hypospadias development.

## Materials and methods

### Subjects

Our study population has been previously described^5^. Boys with second- or third-degree hypospadias and unaffected controls were recruited at Arkansas Children’s Hospital, a region where incidence of hypospadias is increasing^14^. Cases were identified as males undergoing hypospadias-corrective surgery and unaffected males were infants undergoing circumcision at 10.1 and 10.7 months of age, on average, respectively. All subjects reported non-Hispanic white ancestry. Hypospadias was classified based on degree of severity by one of three pediatric urologists at the institution: (1) second-degree hypospadias was defined as a urethral opening in the mid penile to subcoronal area, and (2) third-degree hypospadias was classified based on a urethral opening in the perineum to proximal penile area. For consistency with other reports of hypospadias, we refer to second-degree cases as moderate and third-degree cases as severe. The study protocol was approved by the University of Arkansas for Medical Sciences Institutional Review Board and carried out in accordance with all relevant guidelines and regulations. Informed consent was obtained from all parents and/or legal guardians.

As previously reported^5^, preputial tissue was collected to measure both DNA methylation and genetic variation. For all subjects preputial specimens were collected at the time of surgery and preserved in liquid nitrogen within 30 min from the time of harvest in the operating room. Tissue samples were stored at − 80 °C at the Arkansas Center for Birth Defects Research and Prevention DNA Bank. DNA was extracted from a 1–2 mm section of the frozen tissue shredded with a scalpel prior to processing for DNA isolation using the PerkinElmer Prepito Tissue10 kit according to the manufacturer’s protocol.

### DNA methylation and quality control

DNA methylation of preputial tissue was measured at the Avera Institute for Human Genetics using the Illumina Infinium MethylationEPIC BeadChip (San Diego, CA). All data processing, analyses, and graphics were performed using R^15^. Quality control was performed using the *minfi* package^16^. Samples were filtered to have detection P > 0.01 and one imputed sex mismatch was excluded from the control group. Probes were filtered out that had < 3 beads in ≥ 5% of total samples, non-CpG probes, and SNP-associated probes. After quality control, methylation beta values were normalized using NOOB^17^, which performs background correction and dye-bias normalization. Cell type mixture of preputial tissue was estimated using the *EpiDISH* package^18^ which accounts for epithelial, fibroblast, and blood cell (B cells, NK cells, CD4 T cells, monocytes, neutrophils, and eosinophils) proportions in samples of mixed tissue types. To control for unmeasured batch effects in EWAS, we estimated surrogate variables (SVs) using the *SmartSVA* package^19^. Methylation principal components (mePCs) were estimated using the *irlba* package.

### Genotyping and quality control

DNA concentration was determined by use of the Qubit fluorometer and the Qubit dsDNA BR Assay kit (Life Technologies). Genotyping was performed at the Avera Institute for Human Genetics on the Infinium Global Screening Array (Illumina, San Diego, CA). Quality control methods and imputation for these genotype data are previously reported^5^.

### Epigenome-wide association analyses of hypospadias

We performed three EWAS using a hypospadias case–control study design and two case-only studies: (1) hypospadias cases compared to unaffected controls, (2) hypospadias cases with chordee compared to cases without chordee, and (3) severe hypospadias cases compared to moderate hypospadias cases. All EWAS were analyzed using the R package *CpGassoc*^20^*.* We assessed SVs, mePCs, estimated cell types, tissue sample age, and subject age as technical covariates and adjusted each EWAS model as appropriate to best control the genomic inflation. We corrected for multiple comparisons within each EWAS to identify suggestive associations using the false discovery rate (FDR). Due to the limited sample sizes of this rare outcome in each respective analysis, we selected CpGs from the larger hypospadias case–control EWAS with FDR < 0.2 (N = 91) and CpGs from the chordee and severity case-only EWAS with FDR < 0.15 (N = 45). We elected a lower FDR threshold in the case-only analyses to limit false positives that could be driven by smaller comparison groups. Each of the following functional assessments were additionally performed to validate CpGs suggestively associated with the development of hypospadias.

### Assessment of previous CpGs associated with hypospadias

Within our hypospadias case–control EWAS, we assessed the association of 14 CpGs measured on the Illumina 450 k methylation array previously reported by Choudhry et al^13^. Since CpG sites on the older 450 k array are not entirely captured on the current EPIC array, a complete comparison between the two arrays is not possible. We therefore assessed the Choudhry CpGs for (1) presence on the EPIC array, (2) performance in our quality control pipeline, and (3) association with hypospadias.

### Methylation quantitative trait loci analyses

Methylation at any particular CpG may be influenced by surrounding genetic variation. Methylation within regions identified by GWAS may functionally link reported genetic associations with a phenotype. Additionally, genetic variants predictive of methylation can also serve as instrumental variables and facilitate causal testing in epidemiologic studies. Therefore, to further validate our findings we performed chromosome-wide methylation quantitative trait loci (meQTL) analyses for CpGs suggestively associated with hypospadias. For statistical stability, we transformed normalized methylation beta values to methylation M values. For each CpG with suggestive association in an EWAS, we assessed the association of each single nucleotide polymorphism (SNP) on the same chromosome using SNPTEST v2.5.4^21^ and adjusted meQTL models for mePCs. We used P < 1e−3 and r^2^ < 0.2 to create independent sets of meQTLs for each EWAS CpG.

### Methylation-mediated genetic associations

Methylation within genetic regions previously associated with a phenotype may be causally related to that trait since genetic variation is fixed at conception and methylation fluctuates throughout life. To characterize the potential role of methylation in previously known genetic associations of hypospadias, we identified 22 GWAS regions as those reported by Geller et al.^10^. We then cross-referenced suggestively-associated CpGs to the 500 kb up- and downstream region of each GWAS index variant. For any suggestive CpG mapped to a GWAS region, we then assessed the linkage disequilibrium of our meQTL estimates with the Geller index variant using the 1,000 Genomes CEU reference panel.

### Two-stage least squares Mendelian randomization

The influence of genetic variation on methylation can be leveraged for use as instrumental variables in the causal assessment of CpGs that are measured simultaneously with a health outcome, i.e., when methylation could be the cause or consequence of the phenotype. To assess a causal relationship of DNA methylation on hypospadias development, we performed Mendelian randomization using two-stage least squares Mendelian randomization (2SLS MR). This approach uses individual-level genotype data as instrumental variables and is appropriate for assessing causality in a one-sample Mendelian randomization study design^22^. For each CpG suggestively associated with hypospadias we used independent meQTLs as instrumental variables (IVs) to perform 2SLS MR using the R package *sem*. In the first step, we fitted regression values of methylation M values on a hypospadias outcome, as appropriate for the EWAS from which the CpG was identified, via dosage genotypes of the IVs. In the second step, the hypospadias outcome is estimated from the predicted methylation values from the first step. We defined a significant 2SLS MR test of causal relationship between a CpG and hypospadias outcome as P < 0.05.

### Causal testing for gene expression related to hypospadias

DNA methylation is one type of epigenetic mechanism that regulates gene expression. We sought to characterize the genes within methylation regions identified in our EWAS and further perform causal assessments of those genes with hypospadias in independent datasets. We assessed gene transcripts that may be causally related to hypospadias development using independent publicly available genome-wide association summary data available in the MR-Base web application^23^ to perform two-sample Mendelian randomization. To identify transcripts that may plausibly be regulated by DNA methylation identified in our EWAS, we mapped all UCSC gene symbols within 500 kb up or downstream from the CpG genomic position (a *cis* regulatory region around a CpG). We then identified transcripts across all available tissue types in the Genotype-Tissue Expression (GTEx) project^24^. For each transcript a single expression-associated variant was then used as the IV to assess transcription related to hypospadias. Hypospadias outcome data were extracted from UK Biobank data available from Ben Elsworth at the MRC Integrative Epidemiology Unit in the University of Bristol. The UK Biobank summary data include 26 cases of balanic hypospadias (Q54.0), 10 cases of penile hypospadias (Q54.1), and 39 cases of unspecified hypospadias (Q54.9, i.e., hypospadias that does not conform to the former two classifications) based on ICD10 medical record coding. Genome-wide summary data were available separately for each hypospadias classification and cases were compared to non-cases among a total sample of 463,010 individuals of European ancestry. We defined a significant causal relationship as P < 0.05 for the Wald test comparing the IV effect size between the gene transcript and hypospadias.

## Results

Hypospadias cases from Arkansas comprised 12 moderate cases with chordee, 24 moderate cases without chordee, and 9 severe cases with chordee (Table 1). There were 816,995 CpG sites that passed quality control and were normalized to assess for association with hypospadias. Methylation-estimated proportions of fibroblasts, epithelial cells, and neutrophils differed between preputial tissue from hypospadias cases and unaffected controls (P < 0.05, Supplementary Table S1), but did not differ within cases between chordee and severity. We identified inflation in models not adjusted for technical covariates. In the hypospadias EWAS we adjusted for SVs to correct for differences between cases and controls, estimated cell types, mePCs, subject age, and tissue age. In the chordee EWAS we adjusted for SVs, mePCs, and tissue age. In the severity EWAS we adjusted for SVs, mePCs, and subject age. In methylome-wide analyses we identified differential methylation at 10 CpGs between hypospadias cases and controls (FDR < 0.20), 9 CpGs differentially methylated with chordee (FDR < 0.15), and 6 CpGs differentially methylated by severity (FDR < 0.15) (Table 2). Within the case-only analyses, we observed more CpGs associated with chordee and severity at FDR < 0.2 than were associated with hypospadias in the larger case–control study, therefore we chose a more stringent suggestive FDR threshold (FDR < 0.15) to limit false positive findings in the smaller case-only comparison groups. We then related genetic variation to each suggestive CpG and identified 2–23 independent meQTLs on the same chromosome (Table 2). We used meQTLs as genetic instrumental variables in one-sample 2SLS MR and found support for a causal relationship with hypospadias for 21 of the 25 CpGs identified through our three EWAS studies (Table 2). We further assessed causal relationships of genes in *cis* to these methylation regions and mapped 259 genes measured in multiple tissues in GTEx. Two-sample MR with hypospadias in the UK Biobank indicated that 97 genes neighboring these methylation regions may be causally related to hypospadias (Supplementary Table S2, Figs. S1–S4).

**Table 1:** Characteristics of the hypospadias cases included in epigenome-wide association analyses, shown as n (%) by hypospadias severity and chordee presence.

	Hypospadias cases, N = 45			
	Chordee present, n = 21		Chordee absent, n = 24	
**Moderate, n = 36**				
Distal	6	(13.3)	17	(37.8)
Midshaft	6	(13.3)	6	(13.3)
Subcoronal	0	(–)	1	(2.2)
**Severe, n = 9**				
Penoscrotal	7	(15.6)	0	(–)
Perineal or proximal	2	(4.4)	0	(–)

**Table 2:** Results of the hypospadias case–control and case-only epigenome-wide association studies (EWAS) and two-stage least squares Mendelian randomization (2SLS MR) using methylation quantitative trait loci as instrumental variables for causal association of the CpG with hypospadias characteristics.

EWAS	CpG	Chr	Pos	UCSC Gene	Case^a^ mean beta	Comparison^b^ mean beta	Delta beta	EWAS P-value	EWAS FDR Q-value	n meQTLs	2SLS MR P-value
Hypospadias	cg04714159	1	47,301,259	*CYP4B1-CYP4Z2P*	0.875	0.913	0.038	9.3E−07	0.109	13	3.9E−06
	cg14436889	1	108,231,706	*VAV3*	0.591	0.748	0.157	5.4E−07	0.109	4	9.8E−15
	cg26143053	2	3,718,125	*ALLC*	0.905	0.927	0.023	7.5E−07	0.109	12	0.003
	cg03368481	2	76,014,479	*EVA1A-MRPL19-GCFC2*	0.773	0.847	0.074	8.8E−07	0.109	12	0.191
	cg25918138	3	38,408,147	*XYLB*	0.926	0.944	0.018	1.3E−06	0.132	12	0.001
	cg14906547	9	33,908,335	*UBE2R2*	0.920	0.944	0.025	7.4E−07	0.109	13	8.8E−06
	cg25196688	12	3,421,025	*TSPAN9-PRMT8-EFCAB4B*	0.392	0.506	0.114	1.7E−07	0.109	13	5.5E−10
	cg00752630	12	125,591,239	*AACS*	0.884	0.920	0.037	2.1E−06	0.175	11	0.159
	cg08539758	17	57,638,482	*YPEL2-DHX40-PTRH2*	0.732	0.701	− 0.031	6.0E−07	0.109	5	1.7E−05
	cg26638975	19	51,876,721	*NKG7*	0.780	0.754	− 0.026	2.1E−06	0.175	8	0.025
Chordee	cg07999371	1	26,847,004	*DHDDS-HMGN2-RPS6KA1*	0.558	0.576	0.018	8.5E−07	0.148	20	0.093
	cg05045951	2	70,485,377	*PCYOX1*	0.024	0.021	− 0.003	1.6E−06	0.148	10	5.2E−04
	cg15945209	2	133,039,143	*ANKRD30BL-GPR39*	0.575	0.544	-0.031	5.5E-07	0.148	21	1.3E-07
	cg24241688	3	13,036,636	*IQSEC1*	0.097	0.085	− 0.012	3.3E-07	0.135	15	0.004
	cg06484075	4	184,393,241	*CDKN2AIP-ING2*	0.552	0.592	0.040	1.4E−06	0.148	12	0.011
	cg19445285	12	32,480,897	*BICD1*	0.907	0.886	− 0.020	1.6E-06	0.148	4	1.2E-08
	cg07509211	14	54,950,145	*GMFB*	0.745	0.708	− 0.038	1.4E−07	0.114	2	1.3E−05
	cg16752029	16	27,214,750	*JMJD5* (currently *KMD8*)	0.039	0.043	0.004	1.2E−06	0.148	4	0.041
	cg15014976	19	52,101,661	*SIGLEC6-ZNF175*	0.875	0.862	− 0.013	1.4E−06	0.148	3	0.002
Degree	cg15231374	1	160,132,381	*ATP1A4*	0.663	0.684	0.022	7.0E−07	0.119	13	0.079
	cg22788109	1	225,867,346	*ENAH-SRP9*	0.814	0.747	− 0.067	8.9E−08	0.041	23	6.3E−10
	cg21454600	3	18,772,063	*SATB1-KCNH8*	0.875	0.894	0.020	9.4E−07	0.128	7	3.8E−04
	cg15242360	6	39,854,530	*DAAM2*	0.841	0.872	0.031	7.3E−07	0.119	5	1.0E−04
	cg01749436	8	9,756,178	*TNKS-MSRA*	0.056	0.039	− 0.017	4.2E−07	0.114	14	0.001
	cg10817615	11	45,687,277	*CHST1*	0.523	0.461	− 0.062	1.0E−07	0.041	5	1.9E−09

*2SLS MR* two-stage least squares Mendelian randomization; *Chr* chromosome; *CpG* cytosine-guanine dinucleotide; *EWAS* epigenome-wide association study; *FDR* false discovery rate; *meQTLs* methylation quantitative trait loci; *Pos* genomic position in build 37.

^a^The case groups are: 45 hypospadias cases for the hypospadias case–control study, 21 hypospadias cases with chordee for the chordee case-only study, and 9 severe hypospadias cases for the degree case-only study.

^b^The comparison groups are: 46 boys without hypospadias for the hypospadias case–control study, 24 hypospadias cases without chordee for the chordee case-only study, and 36 moderate hypospadias cases for the degree case-only study.

The strongest association of methylation with hypospadias was identified at *VAV3* cg14436889, with 15.7% less methylation measured in cases compared to controls (P = 5.4 × 10^–7^). Similarly large effects were identified in case–control analyses for cg25196688 (*TSPAN9-PRMT8-EFCAB4B*), where methylation was 11.4% lower in cases compared to controls (P = 1.7 × 10^–7^). The largest difference in methylation between cases with and without chordee was identified at cg06484075 (*WWC2-CLDN22-CDKN2AIP-ING2*), where 4.0% lower methylation was measured in cases with chordee (P = 1.4 × 10^–6^). Higher methylation among cases with chordee was observed for *GMFB* cg07509211 than for cases without chordee (delta beta = 3.8%, P = 1.4 × 10^–7^). When comparing methylation levels by severity, the strongest effect observed was 6.7% higher methylation among severe cases at cg22788109, located between *ENAH* and *SRP9* on chromosome 17 (P = 8.9 × 10^–8^).

We did not replicate association of any CpGs previously associated with hypospadias. Of the 14 CpGs reported by Choudhry et al., two CpGs were not present on the EPIC array, five CpGs did not pass our quality control procedures, and the remaining seven CpGs had an uncorrected P > 0.05 in our case–control analyses (Supplementary Table S3). To identify methylation as a potential mediator of genetic associations with hypospadias, we cross-referenced CpGs identified in our three EWAS to genetic regions identified in the Geller et al. GWAS^10^. We identified methylation at cg15242360 as associated with hypospadias severity, which is located in the *DAAM2* region. However, the rs417096 index variant is not in linkage with meQTLs we identified for this CpG (1,000 Genomes CEU r^2^ ≤ 0.021; Supplementary Fig. S2) and we did not identify direct association of this variant with methylation at cg15242360 in our data (P = 0.07).

## Discussion

Among a sample of 45 moderate to severe cases and 46 controls, we identified 25 DNA methylation sites with evidence of causal association with hypospadias. Additionally, we provide evidence that 97 genes plausibly regulated by these methylation regions may be causally related to hypospadias. Functional annotation of significant genes reveals these methylation regions may be related to hypospadias through altered signaling throughout the course of urogenital development, beginning with formation of the genital tubercle.

**Figure 1:**
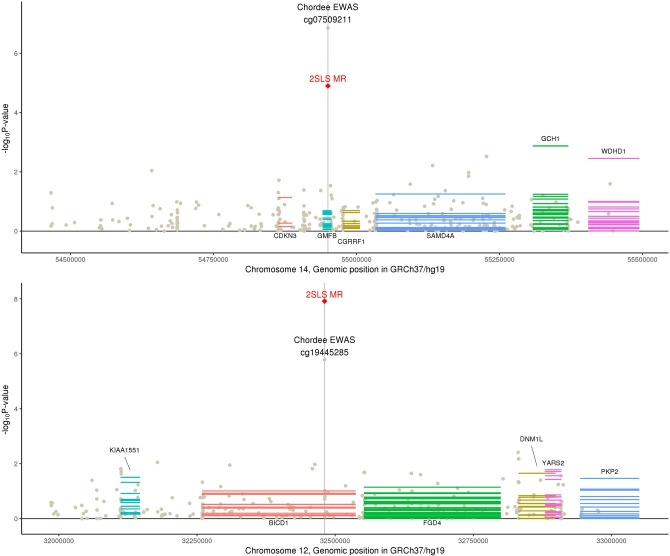
DNA methylation associated with chordee in genic regions involved in differentiation of the germ layers. The x-axis corresponds to genomic position within a chromosome and the y-axis plots − log10 p-values for three sources of statistical testing: (1) a scatter plot for the epigenome-wide association study (EWAS) where the top-associated CpG is indicated by a central vertical line, (2) a single red diamond at the top-associated CpG for two-stage least squares regression Mendelian randomization (2SLS MR) for causal relationship with hypospadias, and (3) horizontal lines across the length of gene transcripts for their causal association with hypospadias where multiple lines represent each tissue type in GTEx. Gene names above the plotted transcripts indicate Wald MR P < 0.05 and gene names below the plotted transcripts indicate Wald MR P > 0.05. Graph was generated by the authors using R version 3.5.2 (https://www.R-project.org/)^15^.

The genital tubercle forms from all three embryonic germ layers and first develops in a sex-independent outgrowth stage, then sexually differentiates dependent on androgen levels^25^. Most of the genital tubercle is composed of mesoderm that develops into erectile tissue and stroma, while the ectoderm develops into the glans penis and skin and the endoderm develops into the urethral epithelium^25,26^.Two genes related to germ layer differentiation were identified within regions of two CpGs differentially methylated with chordee (Fig. 1). Within the *cis*-flanking region of cg07509211, we identified significant causal relationship of hypospadias with expression of *WDHD1,* a WD repeat and HMG-box DNA binding protein that acts as a transcription factor in the mesodermal commitment pathway. We additionally identified *DNM1L* in the region of cg19445285. *DNM1L* is a dynamin GTPase involved in apoptosis during endodermal differentiation and related to beta-catenin signaling^27^. Notably, we identified evidence of causal association of both CpGs with the development of chordee within hypospadias cases, a curvature of the penis that results from skin tethering or fibrosis of other tissues on the ventral side of the penis. We also identified cg25196688 and expression of *TULP3* (TUB like protein 3) as causally related to hypospadias. Sonic hedgehog signaling occurs in the urethral epithelium to control development of the genital tubercle^28^ and TULP3 is necessary for proper ciliary function at this stage^29,30^.

**Figure 2:**
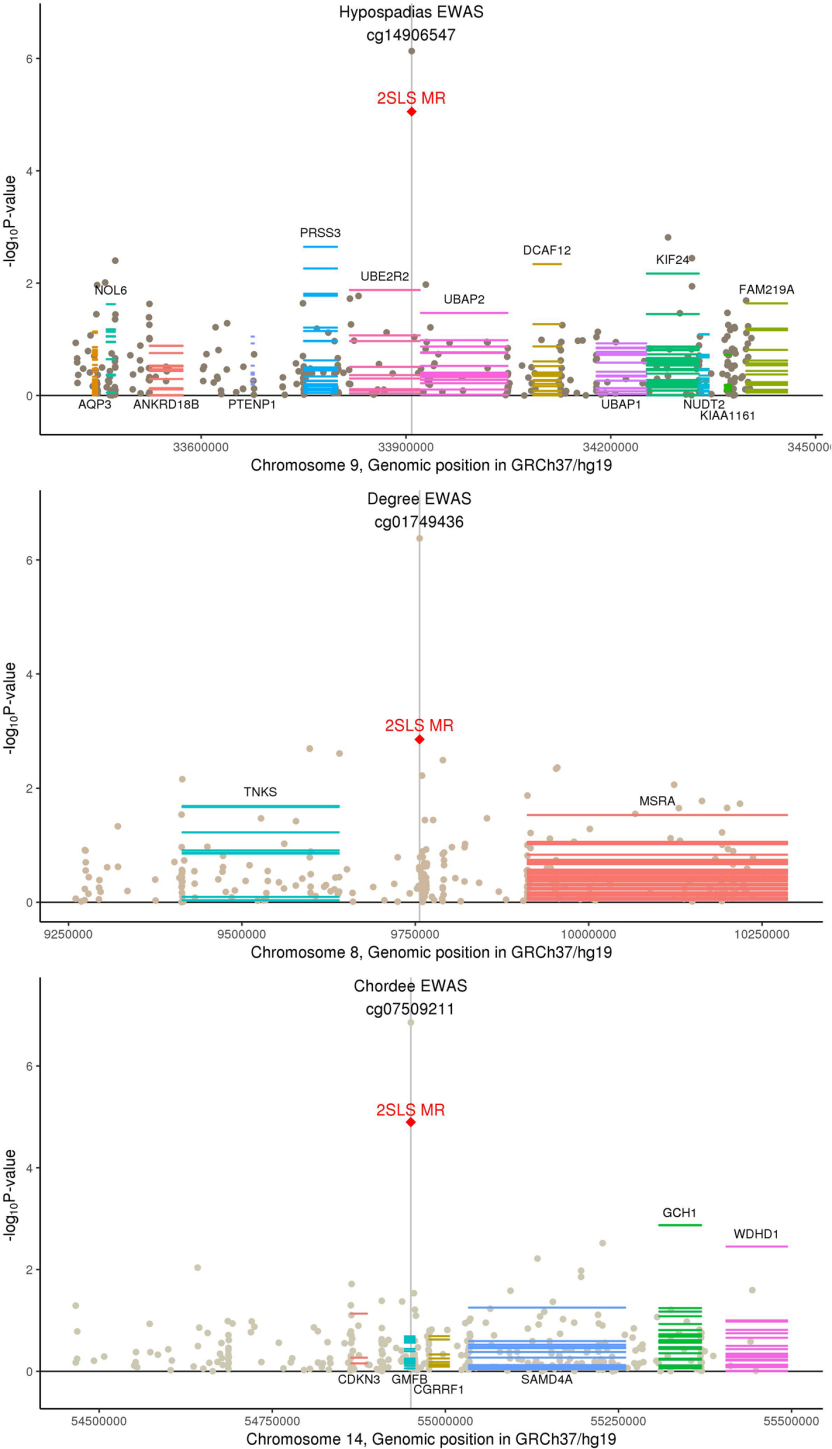
DNA methylation associated with characteristics of hypospadias in genic regions involved in beta-catenin signaling. The x-axis corresponds to genomic position within a chromosome and the y-axis plots − log10 p-values for three sources of statistical testing: (1) a scatter plot for the epigenome-wide association study (EWAS) where the top-associated CpG is indicated by a central vertical line, (2) a single red diamond at the top-associated CpG for two-stage least squares regression Mendelian randomization (2SLS MR) for causal relationship with hypospadias, and (3) horizontal lines across the length of gene transcripts for their causal association with hypospadias where multiple lines represent each tissue type in GTEx. Gene names above the plotted transcripts indicate Wald MR P < 0.05 and gene names below the plotted transcripts indicate Wald MR P > 0.05. Graph was generated by the authors using R version 3.5.2 (https://www.R-project.org/)^15^.

Beta-catenin is a protein that contributes to cell development in the Wnt signaling pathway and is required at multiple stages of formation of the genital tubercle and during urethral development^31,32^. Animal models show that knockout of beta-catenin leads to severe hypospadias^31^ and that estrogen and androgen levels alter beta-catenin signaling to affect penile development^33^. We identified evidence that methylation at cg14906547 (hypospadias), cg07509211 (chordee), and cg01749436 (severity) are causally associated with hypospadias development and its characteristics (Fig. 2). Within these regions, we further identified that expression of genes regulating beta-catenin are related to hypospadias, including *UBE2R2*^34^ (ubiquitin-conjugating enzyme), *PKP2*^35^ (plakophilin), and *TNKS*^36^ (tankyrase).

**Figure 3:**
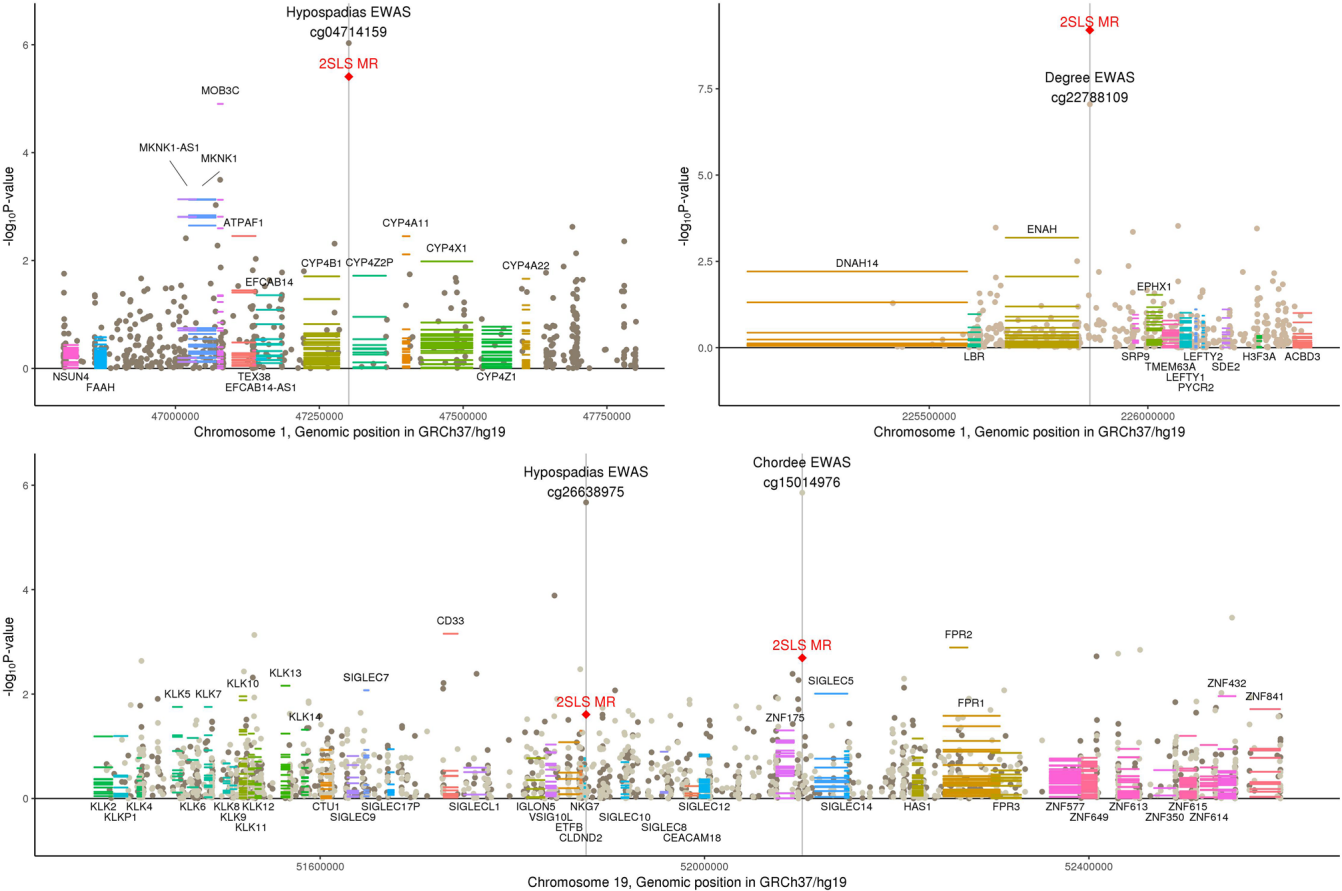
DNA methylation associated with characteristics of hypospadias in genic regions related to androgens. The x-axis corresponds to genomic position within a chromosome and the y-axis plots − log10 p-values for three sources of statistical testing: (1) a scatter plot for the epigenome-wide association study (EWAS) where the top-associated CpGs are each indicated by a vertical line, (2) a single red diamond at each top-associated CpG for two-stage least squares regression Mendelian randomization (2SLS MR) for causal relationship with hypospadias, and 3) horizontal lines across the length of gene transcripts for their causal association with hypospadias where multiple lines represent each tissue type in GTEx. Gene names above the plotted transcripts indicate Wald MR P < 0.05 and gene names below the plotted transcripts indicate Wald MR P > 0.05. Two CpGs in the same region are depicted in the third panel, cg26638975 from the hypospadias case–control EWAS (dark brown scatter points) and cg15014976 from the chordee case-only EWAS (light brown scatter points). Graph was generated by the authors using R version 3.5.2 (https://www.R-project.org/)^15^.

In addition to their effect on beta-catenin signaling, sex steroids regulate differentiation of the genital tubercle into the male penis. Hormonal disruptions during penile development can cause urethral and epithelial abnormalities that result in hypospadias and chordee^37,38^. The cytochrome P450 superfamily of enzymes are responsible for catalyzing the synthesis of sex steroids from cholesterol, fatty acids, and other lipids. We identified a significant causal effect of methylation at cg04714159 on hypospadias, as well as potential causal effects of multiple members of the cytochrome P450 family of enzymes in this region (*CYP4A11, CYP4A22, CYP4B1, CYP4X1, CYP4Z2P*; Fig. 3). We additionally identified a causal association of methylation at cg22788109 and expression of *EPHX1* with hypospadias, which encodes an epoxide hydrolase involved in the cytochrome P450 metabolism of epoxide-containing fatty acids^39^. Additionally, these genes have been implicated in pre-eclampsia, a gestational blood pressure disorder that is consistently associated with hypospadias in epidemiologic studies^40^. CYP4A11, CYP4A22, and other cytochrome P450 isoforms are differentially expressed in preeclamptic placentas^41^ and *EPHX1* mutations contribute to preeclampsia^42^.

Further support for the involvement of sex steroids in hypospadias development is evidenced by two likely causal associations of cg26638975 and cg15014976 with hypospadias and chordee, respectively (Fig. 3). This region on chromosome 19 is characterized by clusters of sialic acid-binding Ig-like lectin (*SIGLEC*) and kallikrein (*KLK*) genes, among which we identified likely causal relationships with hypospadias (*CD33/SIGLEC3, SIGLEC5, SIGLEC7, KLK5, KLK7, KLK10, KLK13*, and *KLK14*). The *KLK* family is involved in the kinin system, a hormonal pathway that regulates blood pressure, and *KLK* expression is upregulated by sex steroids^43,44^. The two methylation signals observed in the *SIGLEC-KLK* region are not strongly correlated (Supplementary Fig. S1) and have independent meQTLs (1,000 Genomes CEU r^2^ ≤ 0.022; Supplementary Fig. S2), thus cg26638975 and cg15014976 may represent two independent regulatory mechanisms in this region. An additional gene of interest in this region is *SIGLEC6* that binds the obesity-associated leptin molecule. Expression of *SIGLEC6* is consistently upregulated in the placentas of women with preeclampsia^45–49^, as well as with the presence of other placental abnormalities^50^. However, *SIGLEC6* had a single tissue-specific eQTL estimate in GTEx that was not present in the UK Biobank, therefore we were not able to test a causal relationship with hypospadias.

**Figure 4:**
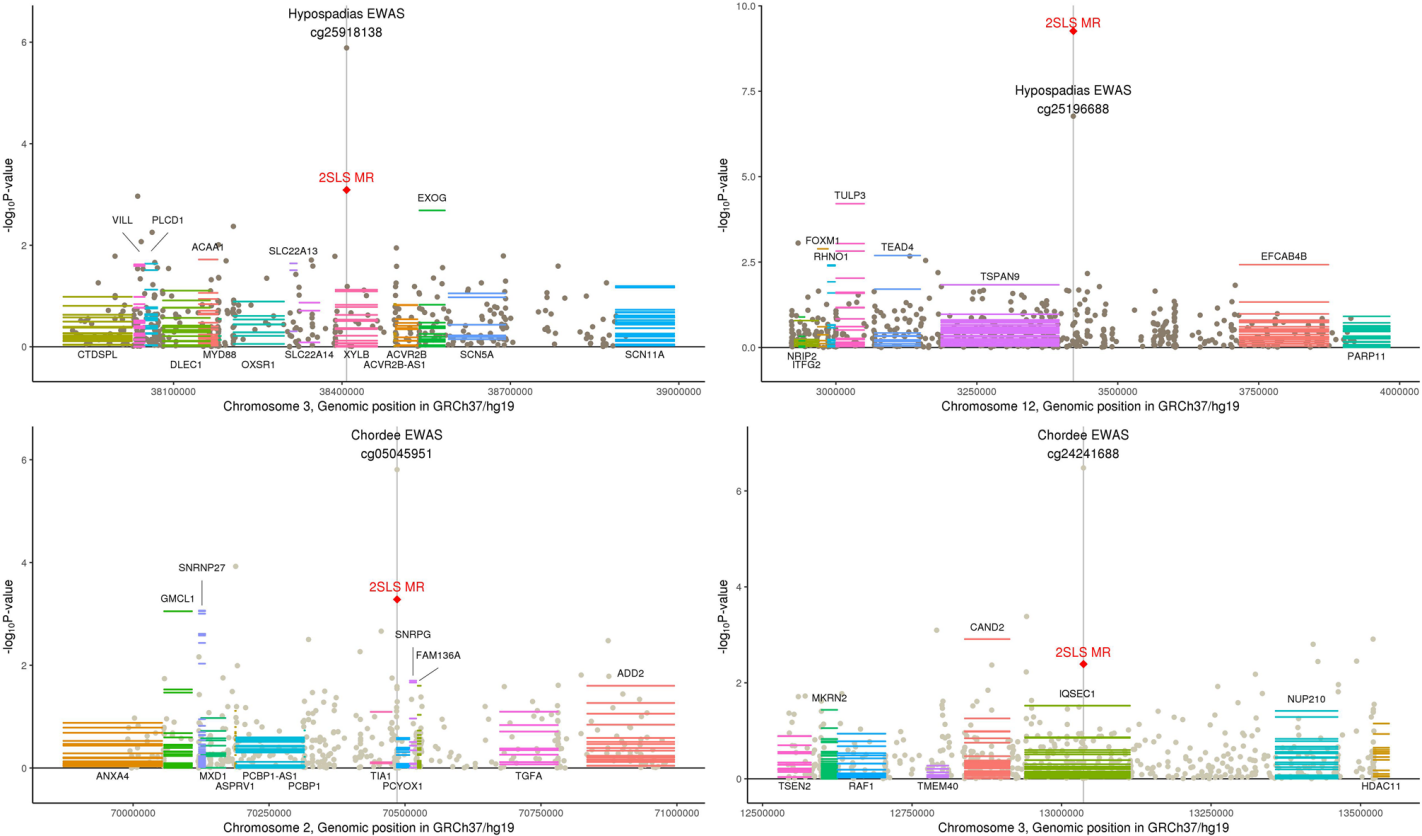
DNA methylation associated with characteristics of hypospadias in genic regions related to reproductive traits. The x-axis corresponds to genomic position within a chromosome and the y-axis plots − log10 p-values for three sources of statistical testing: (1) a scatter plot for the epigenome-wide association study (EWAS) where the top-associated CpG is indicated by a central vertical line, (2) a single red diamond at the top-associated CpG for two-stage least squares regression Mendelian randomization (2SLS MR) for causal relationship with hypospadias, and (3) horizontal lines across the length of gene transcripts for their causal association with hypospadias where multiple lines represent each tissue type in GTEx. Gene names above the plotted transcripts indicate Wald MR P < 0.05 and gene names below the plotted transcripts indicate Wald MR P > 0.05. Graph was generated by the authors using R version 3.5.2 (https://www.R-project.org/)^15^.

We additionally identified methylation regions associated with hypospadias where gene transcripts are related to reproductive traits (Fig. 4). We identified cg25918138, cg25196688, cg05045951, and cg24241688 as causally related to hypospadias and its characteristics. Genes in these regions related to male fertility include *ACAA1* (acetyl-CoA acyltransferase 1)^51,52^, *PLCD1* (phospholipase C delta 1)^53^, *EFCAB4B (CRACR2A*; calcium release activated channel regulator 2A)^54^, *GMCL1* (spermatogenesis associated germ cell-less protein expression decreased in male infertility)^55^, and *MKRN2* (makorin ring finger protein 2)^56^. *DNM1L*, discussed previously for implications in germ cell differentiation, is also differentially expressed in sperm with poor motility^57^, and expression of multiple members of the *KLK* family are associated with measures of sperm quality^58^. Additionally, *TEAD4* and *TSPAN9* in the *cis* region of cg25196688 is associated with fallopian tube disease^59^. While infertility may be a long-term consequence among males with hypospadias, we assessed causal direction in these regions and both methylation and gene expression are suggested to be causally related to hypospadias. We postulate that methylation at these CpG sites may be inherited from the parents and reflect fertility of the parents of hypospadias cases. In fact, hypospadias is one type of birth defect more prevalent among families seeking fertility treatments^60,61^.

We were unable to replicate association of 14 CpGs previously reported as associated with hypospadias^13^. Differences in our findings could be due to hypospadias severity (milder cases in Choudhry) or age at preputial tissue collection (an older median 26 months of age in Choudhry), however, the previous EWAS did not report any quality control of the methylation array, normalization, and control for potential batch effects that have become standard procedure for analysis of methylation array data. We additionally assessed whether methylation could mediate previous hypospadias GWAS regions. We did not identify methylation at cg15242360 to mediate the *DAAM2* rs417096 index variant reported in the Geller et al. GWAS^10^. However, we identified causal support for cg15242360 and two gene transcripts in the development of hypospadias. *KIF6* is a kinesin motor protein ubiquitously expressed in coronary and vascular tissues. *MOCS1* is involved in the synthesis of molybdenum cofactor, which metabolizes water-soluble vitamins and other cofactors. Autosomal recessive *MOCS1* mutation can result in an inborn error of metabolism. A case report a female 46,XY patient with testicular dysgenesis identified a large inherited duplication of the *DAAM2-MOCS1* region and notably this proband is reported to have a brother with penoscrotal hypospadias^62^.

A limitation of this study is the dynamic nature of methylation and gene expression levels, particularly as they regulate human development. We measured methylation levels in the preputial tissue of boys at 10 months of age, on average. We also assessed gene expression levels across all tissues in GTEx, for which the samples comprise a variety of ages. Accessing the relevant tissue types at the developmentally-appropriate time period of urethral development in humans is not feasible. Therefore, we are limited to identify methylation or expression levels that remain altered after urethral development and are detectable across multiple or related tissue types. Further, methylation may itself be altered by adverse health conditions, which has been supported by causal testing in multiple cross-sectional EWAS^63^. However, young boys with hypospadias have limited health consequences from their condition, therefore methylation differences are unlikely to be a result of the hypospadias. Also, within the data available, we are not able to functionally relate DNA methylation to gene transcription. Despite these limitations, our study measured methylation in preputial tissue and achieved the greatest statistical power to date to relate methylation to development of this relatively rare health outcome. Our study had adequate statistical power to detect relatively large effect sizes (1.8–15.7% difference in methylation beta values) at sites that remained differentially methylated after embryo development. Through the analysis of DNA methylation among moderate to severe hypospadias cases and gene expression in independent samples, we have identified genetic mechanisms that may be involved in early penile development. Methylation and gene transcripts in these regions should be considered for future functional assessments of human development and morphology.

## Supplementary information

Supplementary figures

Supplementary tables

## Data Availability

The data that support the findings of this study are available from the corresponding author upon reasonable request.

## References

[1] Schnack TH (2008). Familial aggregation of hypospadias: a cohort study. Am. J. Epidemiol..

[2] Carmichael SL, Shaw GM, Lammer EJ (2012). Environmental and genetic contributors to hypospadias: a review of the epidemiologic evidence. Birth Defects Res. A.

[3] Agopian AJ (2016). Maternal hypertension and risk for hypospadias in offspring. Am. J. Med. Genet. A.

[4] Agopian AJ, Langlois PH, Ramakrishnan A, Canfield MA (2014). Epidemiologic features of male genital malformations and subtypes in Texas. Am. J. Med. Genet. A.

[5] Richard MA (2019). The role of genetic variation in DGKK on moderate and severe hypospadias. Birth Defects Res..

[6] Smith ZD, Meissner A (2013). DNA methylation: roles in mammalian development. Nat. Rev. Genet..

[7] Joo JE (2018). Heritable DNA methylation marks associated with susceptibility to breast cancer. Nat. Commun..

[8] Carmichael SL, Witte JS, Ma C, Lammer EJ, Shaw GM (2014). Hypospadias and variants in genes related to sex hormone biosynthesis and metabolism. Andrology.

[9] Carmichael SL (2013). Hypospadias and genes related to genital tubercle and early urethral development. J. Urol..

[10] Geller F (2014). Genome-wide association analyses identify variants in developmental genes associated with hypospadias. Nat. Genet..

[11] Arendt LH (2018). Maternal diabetes mellitus and genital anomalies in male offspring: a nationwide cohort study in 2 Nordic Countries. Epidemiology.

[12] Arendt LH (2017). Maternal overweight and obesity and genital anomalies in male offspring: a population-based Swedish cohort study. Paediatr. Perinat. Epidemiol..

[13] Choudhry S (2012). Genome-wide DNA methylation profiling of CpG islands in hypospadias. J. Urol..

[14] Canon S, Mosley B, Chipollini J, Purifoy JA, Hobbs C (2012). Epidemiological assessment of hypospadias by degree of severity. J. Urol..

[15] *R: A Language and Environment for Statistical Computing* (R Foundation for Statistical Computing, Vienna, Austria, 2018).

[16] Aryee MJ (2014). Minfi: a flexible and comprehensive Bioconductor package for the analysis of Infinium DNA methylation microarrays. Bioinformatics.

[17] Triche TJ, Weisenberger DJ, Van Den Berg D, Laird PW, Siegmund KD (2013). Low-level processing of Illumina Infinium DNA Methylation BeadArrays. Nucleic Acids Res..

[18] Zheng SC, Breeze CE, Beck S, Teschendorff AE (2018). Identification of differentially methylated cell types in epigenome-wide association studies. Nat. Methods.

[19] Chen J (2017). Fast and robust adjustment of cell mixtures in epigenome-wide association studies with SmartSVA. BMC Genom..

[20] Barfield RT, Kilaru V, Smith AK, Conneely KN (2012). CpGassoc: an R function for analysis of DNA methylation microarray data. Bioinformatics.

[21] Marchini J, Howie B (2010). Genotype imputation for genome-wide association studies. Nat. Rev. Genet..

[22] Teumer A (2018). Common methods for performing Mendelian randomization. Front. Cardiovasc. Med..

[23] Hemani G (2018). The MR-Base platform supports systematic causal inference across the human phenome. Elife.

[24] Consortium, G (2015). Human genomics. The Genotype-Tissue Expression (GTEx) pilot analysis: multitissue gene regulation in humans. Science.

[25] Baskin L (2018). Development of the human penis and clitoris. Differentiation.

[26] Cohn MJ (2011). Development of the external genitalia: conserved and divergent mechanisms of appendage patterning. Dev. Dyn..

[27] Ma Y (2019). CHIR-99021 regulates mitochondrial remodelling via beta-catenin signalling and miRNA expression during endodermal differentiation. J. Cell Sci..

[28] Perriton CL, Powles N, Chiang C, Maconochie MK, Cohn MJ (2002). Sonic hedgehog signaling from the urethral epithelium controls external genital development. Dev. Biol..

[29] Norman RX (2009). Tubby-like protein 3 (TULP3) regulates patterning in the mouse embryo through inhibition of Hedgehog signaling. Hum. Mol. Genet..

[30] Qin J, Lin Y, Norman RX, Ko HW, Eggenschwiler JT (2011). Intraflagellar transport protein 122 antagonizes Sonic Hedgehog signaling and controls ciliary localization of pathway components. Proc. Natl. Acad. Sci. USA.

[31] Lin C, Yin Y, Long F, Ma L (2008). Tissue-specific requirements of beta-catenin in external genitalia development. Development.

[32] Miyagawa S (2009). Genetic interactions of the androgen and Wnt/beta-catenin pathways for the masculinization of external genitalia. Mol. Endocrinol..

[33] Chen Y (2018). Hormone-responsive genes in the SHH and WNT/beta-catenin signaling pathways influence urethral closure and phallus growth. Biol. Reprod..

[34] Semplici F, Meggio F, Pinna LA, Oliviero S (2002). CK2-dependent phosphorylation of the E2 ubiquitin conjugating enzyme UBC3B induces its interaction with beta-TrCP and enhances beta-catenin degradation. Oncogene.

[35] Chen X, Bonne S, Hatzfeld M, van Roy F, Green KJ (2002). Protein binding and functional characterization of plakophilin 2. Evidence for its diverse roles in desmosomes and beta -catenin signaling. J. Biol. Chem..

[36] Huang SM (2009). Tankyrase inhibition stabilizes axin and antagonizes Wnt signalling. Nature.

[37] Ma L (2015). Battle of sex hormones in genitalia anomalies. Proc. Natl. Acad. Sci. USA.

[38] Zheng Z, Armfield BA, Cohn MJ (2015). Timing of androgen receptor disruption and estrogen exposure underlies a spectrum of congenital penile anomalies. Proc. Natl. Acad. Sci. USA.

[39] Edin ML (2018). Epoxide hydrolase 1 (EPHX1) hydrolyzes epoxyeicosanoids and impairs cardiac recovery after ischemia. J. Biol. Chem..

[40] Sheriff FR (2019). Maternal hypertension and hypospadias in offspring: A systematic review and meta-analysis. Birth Defects Res..

[41] Plenty NL (2018). Arachidonic acid metabolites of CYP4A and CYP4F are altered in women with preeclampsia. Prostaglandins Lipid Mediat..

[42] Zusterzeel PL (2001). A polymorphism in the gene for microsomal epoxide hydrolase is associated with pre-eclampsia. J. Med. Genet..

[43] Luo LY, Grass L, Diamandis EP (2003). Steroid hormone regulation of the human kallikrein 10 (KLK10) gene in cancer cell lines and functional characterization of the KLK10 gene promoter. Clin. Chim. Acta.

[44] Slagter MH (2006). Effect of testosterone administration on serum and urine kallikrein concentrations in female-to-male transsexuals. Clin. Chem..

[45] Kaartokallio T (2015). Gene expression profiling of pre-eclamptic placentae by RNA sequencing. Sci. Rep..

[46] Trifonova EA (2014). Analysis of the placental tissue transcriptome of normal and preeclampsia complicated pregnancies. Acta Nat..

[47] Rumer KK, Uyenishi J, Hoffman MC, Fisher BM, Winn VD (2013). Siglec-6 expression is increased in placentas from pregnancies complicated by preterm preeclampsia. Reprod. Sci..

[48] Kang JH (2011). Preeclampsia leads to dysregulation of various signaling pathways in placenta. J. Hypertens..

[49] Winn VD (2009). Severe preeclampsia-related changes in gene expression at the maternal-fetal interface include sialic acid-binding immunoglobulin-like lectin-6 and pappalysin-2. Endocrinology.

[50] Rumer KK (2012). Siglec-6 is expressed in gestational trophoblastic disease and affects proliferation, apoptosis and invasion. Endocr. Relat. Cancer.

[51] Mizuno Y (2013). Tysnd1 deficiency in mice interferes with the peroxisomal localization of PTS2 enzymes, causing lipid metabolic abnormalities and male infertility. PLoS Genet..

[52] Amaral A (2013). Human sperm tail proteome suggests new endogenous metabolic pathways. Mol. Cell. Proteom..

[53] Maruyama SY (2016). A critical role of solute carrier 22a14 in sperm motility and male fertility in mice. Sci. Rep..

[54] Krausz C, Escamilla AR, Chianese C (2015). Genetics of male infertility: from research to clinic. Reproduction.

[55] Liu XX, Cai L, Liu FJ (2018). An in silico analysis of human sperm genes associated with asthenozoospermia and its implication in male infertility. Medicine (Baltimore).

[56] Qian X (2016). Deficiency of Mkrn2 causes abnormal spermiogenesis and spermiation, and impairs male fertility. Sci. Rep..

[57] Marchiani S (2014). SUMO1 in human sperm: new targets, role in motility and morphology and relationship with DNA damage. Reproduction.

[58] Emami N (2009). Association between kallikrein-related peptidases (KLKs) and macroscopic indicators of semen analysis: their relation to sperm motility. Biol. Chem..

[59] Nowee ME (2007). DNA profiling of primary serous ovarian and fallopian tube carcinomas with array comparative genomic hybridization and multiplex ligation-dependent probe amplification. J. Pathol..

[60] Arendt LH (2018). Parental subfertility and hypospadias and cryptorchidism in boys: results from two Danish birth cohorts. Fertil. Steril..

[61] Liberman RF (2017). Assisted reproductive technology and birth defects: effects of subfertility and multiple births. Birth Defects Res..

[62] Calvel P (2015). A case of Wiedemann-Steiner syndrome associated with a 46, XY disorder of sexual development and gonadal dysgenesis. Sex. Dev..

[63] Dekkers KF (2016). Blood lipids influence DNA methylation in circulating cells. Genome Biol..

