# Supplementary material for: Altered mechanisms of genital development identified through integration of DNA methylation and genomic measures in hypospadias
Source: Sci Rep. 2020 Jul 29;10:12715. doi: 10.1038/s41598-020-69725-1 (PMC7391634; doi:10.1038/s41598-020-69725-1)
Supplement: Supplementary file 1 — Supplementary figures [file 41598_2020_69725_MOESM1_ESM.docx]

**SUPPLEMENTARY FIGURES:**

**Altered mechanisms of genital development identified through integration of DNA methylation and genomic measures in hypospadias**

Melissa A. Richard, Pagna Sok, Stephen Canon, Wendy N. Nembhard, Austin L. Brown, Erin C. Peckham-Gregory, Minh Ton, Erik A. Ehli, Noah A. Kallsen, Shanna A. Peyton, Gareth E. Davies, Ashay Patel, Ismael Zamilpa, Charlotte A. Hobbs, Michael E. Scheurer, Philip J. Lupo


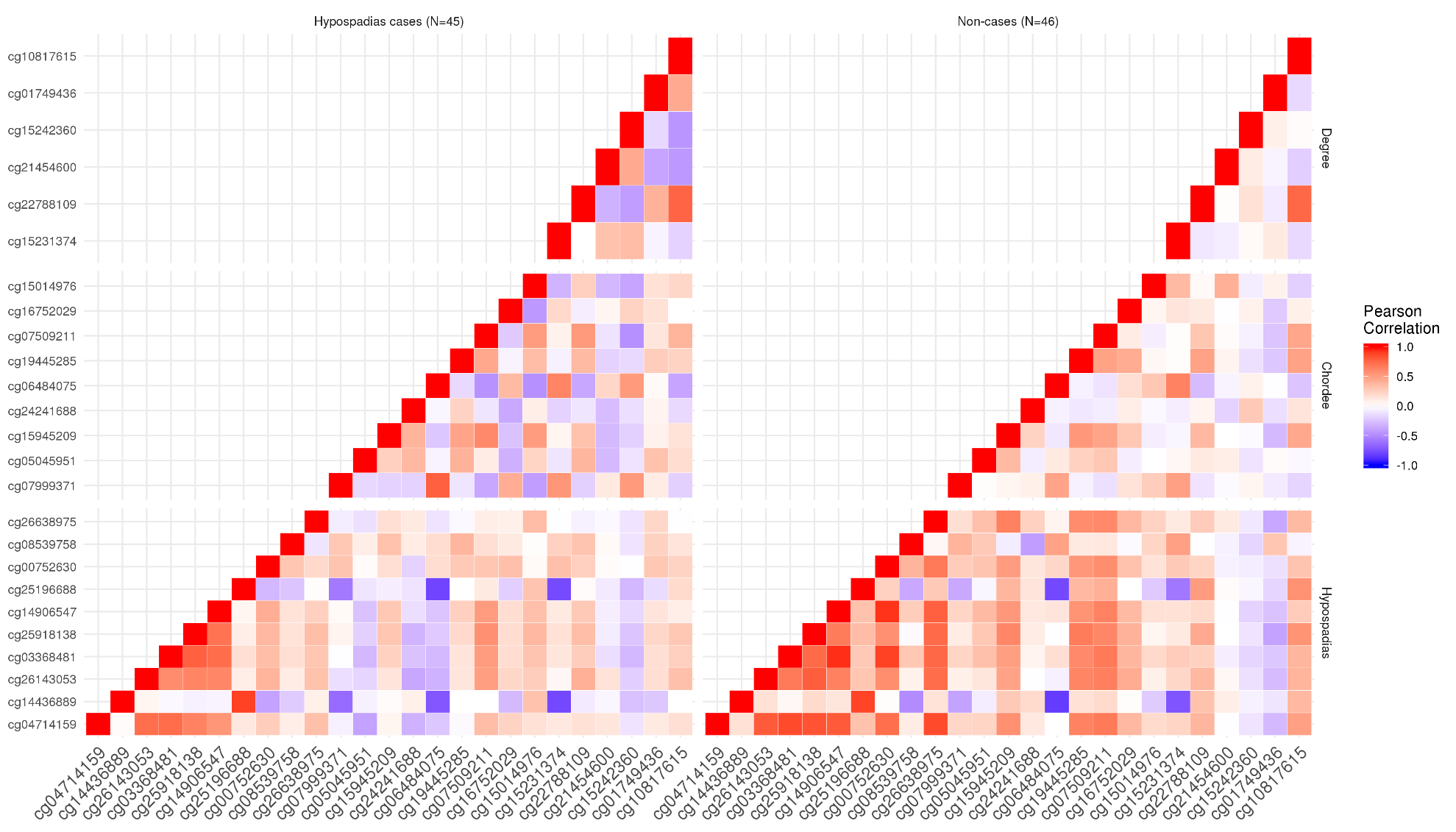


**Supplemental Figure 1**. Pearson correlation patterns of methylation beta values at CpGs suggestively associated with hypospadias, chordee, or degree. Methylation correlation within all hypospadias cases is shown in the left panel and methylation correlation within all non-cases is shown in the right panel.

**
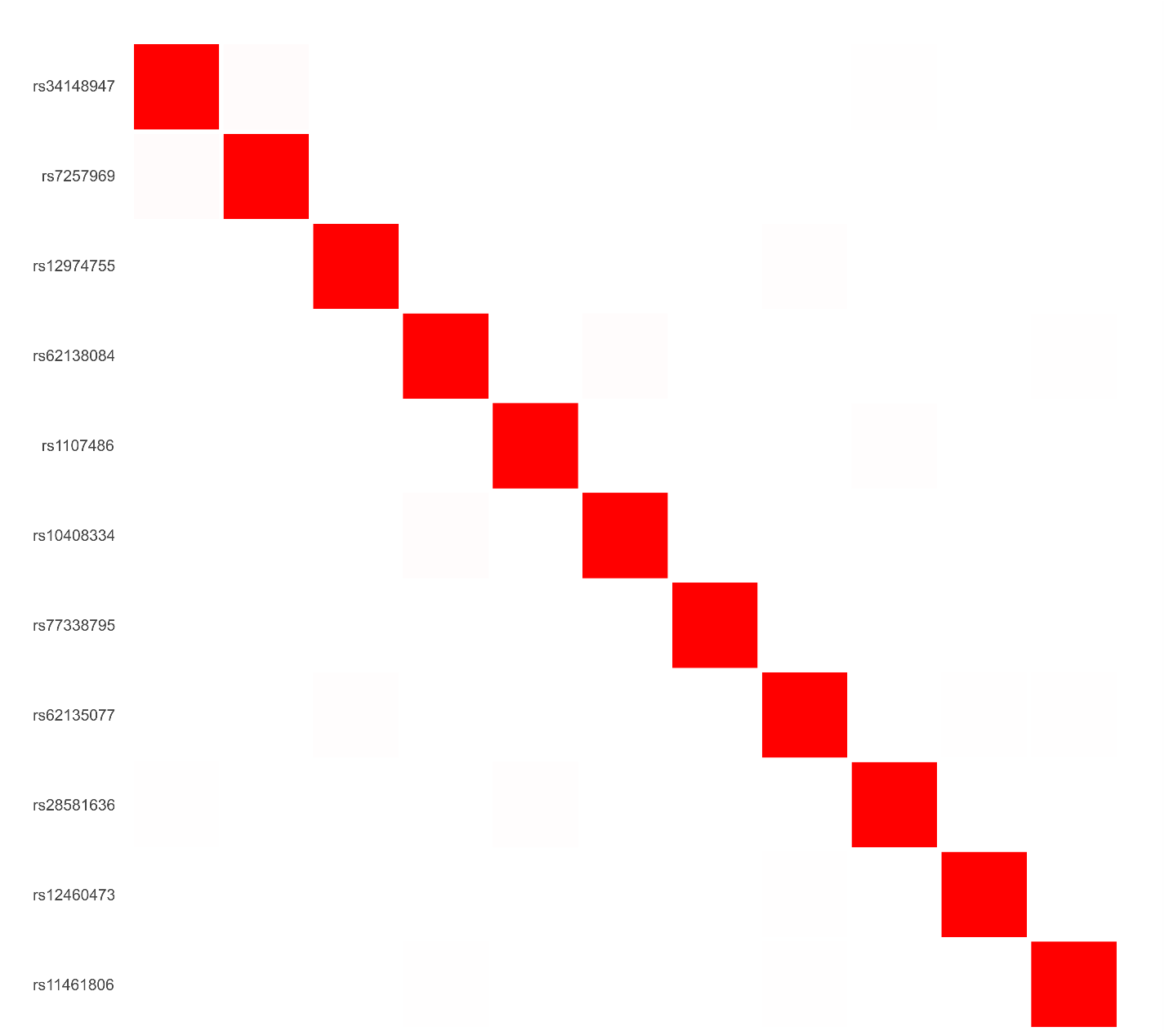
**

**Supplemental Figure 2.** Linkage disequilibrium estimates from 1000 Genomes CEU for methylation quantitative trait loci for cg26638975 and cg15014976, two methylation signals identified for association with hypospadias and chordee, respectively, in the *SIGLEC-KLK* region. All meQTLs are independent with r^2^≤0.022.


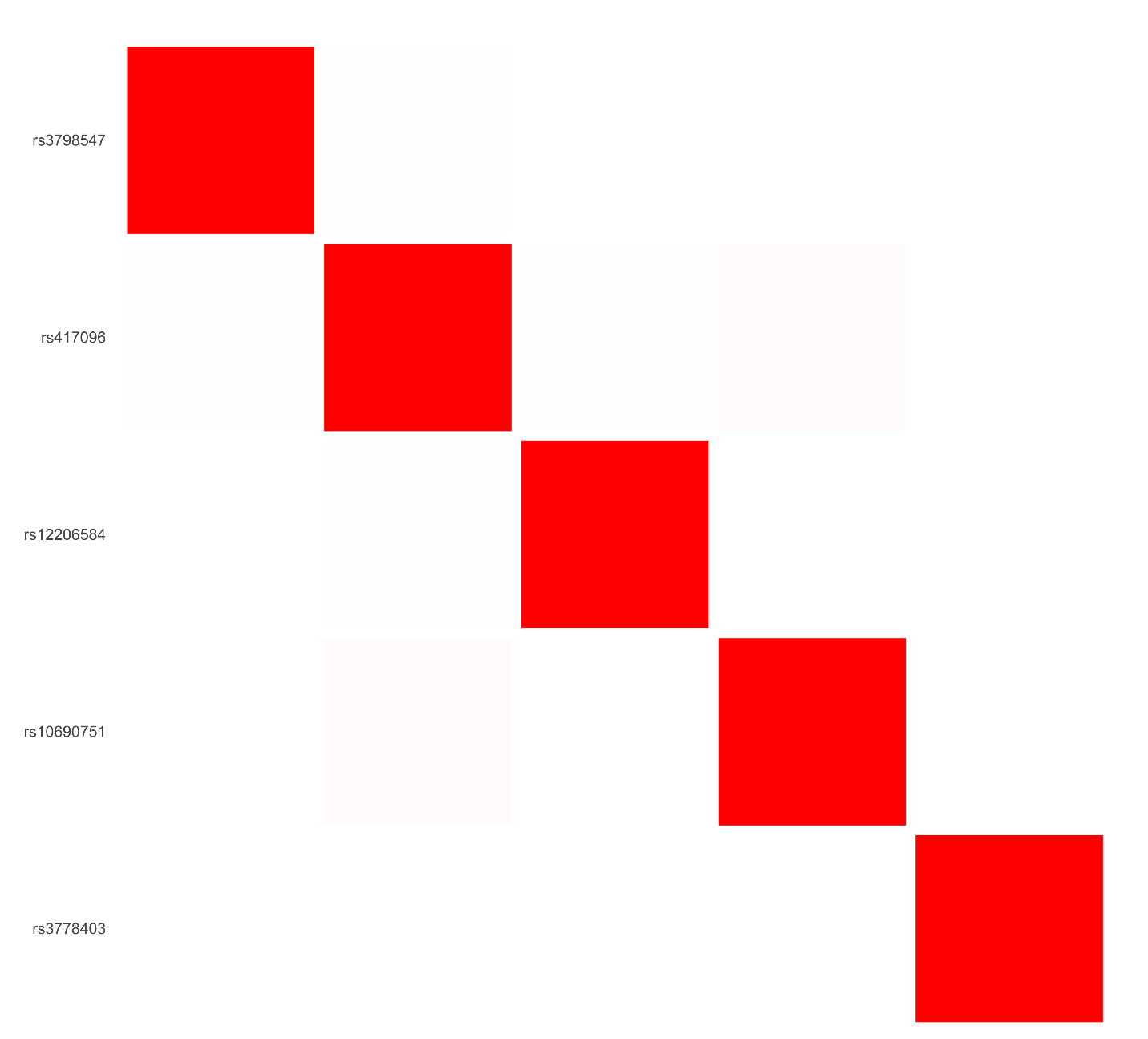


**Supplemental Figure 3.** Linkage disequilibrium estimates from 1000 Genomes CEU for methylation quantitative trait loci (meQTL) for *DAAM2* cg15242360 with the *DAAM2* index variant identified for association with hypospadias by Geller et al (rs417096). All meQTLs and rs417096 are independent with r^2^≤0.021.
